# Single low-dose ketamine infusion for women with prenatal depressive symptoms undergoing cesarean delivery: A pilot randomized trial

**DOI:** 10.3389/fsurg.2022.1050232

**Published:** 2022-12-08

**Authors:** Shuo Wang, Chun-Mei Deng, Yuan Zeng, Jia-Hui Ma, Yuan Qu, Dong-Xin Wang

**Affiliations:** ^1^ Department of Anesthesiology and Critical Care Medicine, Peking University First Hospital, Beijing, China; ^2^ Outcomes Research Consortium, Cleveland, OH, United States

**Keywords:** perinatal care, depressive disorders, anesthesia obstetrical, ketamine, postpartum depression

## Abstract

**Background:**

Ketamine is approved for antidepressant therapy, but evidence regarding its use in women with perinatal depression is lacking. Herein, we investigated the effects of low-dose ketamine in women with prenatal depressive symptoms and tested the feasibility of a future large randomized trial.

**Methods:**

This was a randomized, double-blind, placebo-controlled pilot trial. Sixty-six women with prenatal depressive symptoms who were scheduled for elective cesarean delivery were randomized to receive either low-dose ketamine (0.5 mg/kg) or placebo (normal saline). The study drugs were intravenously infused over a 40-minute period after clamping the umbilical cord. The primary outcome was depression score assessed with the Edinburgh Postnatal Depression Scale at 48 h postpartum. Among other and safety outcomes, occurrence of nausea or vomiting was observed, pain intensity was assessed with the numeric rating scale. The feasibility of implementing the protocol was also evaluated.

**Results:**

A total of 64 parturients were included in the intention-to-treat analysis. The depression score at 48 h did not differ between groups: median 9 (interquartile range 6 to 13) with ketamine vs. 8 (6 to 10) with placebo; median difference 1, 95% CI −1 to 3; *P* = 0.608. The pain intensity at 4 h postpartum was less severe in the ketamine group (median difference −1, 95% CI −1 to 0, *P* = 0.002). Among safety outcomes, intraoperative nausea or vomiting was less common in patients given ketamine (0.0% [0/33] with ketamine vs. 21.2% [7/33] with placebo, *P* = 0.011). Recruitment was satisfactory and the protocol was acceptable to participants and clinicians.

**Conclusions:**

A single low-dose ketamine infusion did not decrease the depression score at 2 days, but reduced intraoperative nausea and vomiting and lowered pain intensity at 4 h after cesarean delivery among women with prenatal depressive symptoms. The study protocol is feasible for a large randomized trial.

**Clinical Trial Registration:**

The study was registered with ClinicalTrials.gov (identifier: NCT03336541; 08/11/2017).

## Introduction

Depression is a common mental disorder among women during the perinatal period. The reported prevalence ranges from 6.9% to 12.9% in high-income countries, but is higher in middle-income and low-income countries with pooled rate of 20.8% and 25.8%, respectively (1–4). The occurrence of perinatal depression is associated with a series of negative consequences on both mothers and offspring such as impaired mother-infant interactions, risky behavior including suicidal and infanticidal ideation, and poor developmental trajectories causing emotional, behavioral, and cognitive problems (5–7). Much attention has been paid to the prevention of perinatal maternal depression. Non-pharmacological measures are helpful; medical intervention is also necessary in some patients yet evidence is limited in this patient population (8, 9).

Ketamine, a non-competitive antagonist of the N-methyl-D-aspartate receptor, has been used as an anesthetic and analgesic drug during surgery for more than half a century. Over the last 20 years, ketamine is found to have antidepressant effect and is approved to treat major depressive disorder and bipolar disorder (10, 11), although the underlying mechanisms are not totally clear (12, 13). There are studies that investigated the effect of ketamine on mood and depression in the perioperative settings, but results remain inconclusive until recently (14, 15).

The effect of ketamine on postpartum depression has been investigated in women undergoing cesarean delivery. In a randomized trial of 330 parturients, intraoperative low-dose ketamine (0.25 mg/kg) did not reduce the prevalence of depression at 3 days and 6 weeks after cesarean delivery (16). In another trial of 654 women, intraoperative ketamine (0.5 mg/kg) decreased the prevalence of postpartum blues at 4 days and the prevalence of postpartum depression at 42 days (17). In a recent trial of 330 healthy women undergoing cesarean delivery, low-dose ketamine (0.25 mg/kg) reduced the prevalence of depressive symptoms at 1 week but not later (18). In the above studies, baseline depression was assessed in only one trial in which only a small proportion (36%) of the enrolled women had prenatal depression (17).

The effect of low-dose ketamine might be more prominent in women with prenatal depression; however, studies are lacking in this patient population. We therefore conducted a pilot randomized trial to evaluate whether a single low-dose ketamine infusion during cesarean delivery could decrease the depression scores at 2 days postpartum in parturients with prenatal depression, and to evaluate the feasibility of a large randomized trial using this protocol.

## Materials and methods

### Study design and ethics

This was a randomized, double-blind, placebo-controlled pilot trial with two parallel-arms. The study protocol was approved by the local Clinical Research Ethics Committee [2017(36); 08/11/2017; principal investigator: D-XW] and was registered with ClinicalTrails.gov (identifier: NCT03336541; 08/11/2017). The trial was conducted in Peking University First Hospital (Beijing, China). Written informed consent was obtained from each participant before collecting data.

### Participant recruitment

We enrolled women aged 18 to 45 years who had a prenatal Edinburgh Postnatal Depression Scale (EPDS) of 10 or higher and were scheduled for elective cesarean delivery. Patients were excluded if they met any of the following criteria: (1) history of psychiatric diseases diagnosed before or during pregnancy by psychiatrists; (2) communication difficulties; (3) presence of contraindications to neuraxial anesthesia, including previous infectious disease of the central nervous system, spinal or intraspinal disease, systemic infection, skin or soft tissue infection at the site of puncture, or coagulopathy; (4) severe pregnancy complications, such as severe preeclampsia, placenta implantation, or HELLP syndrome (a syndrome of intravascular hemolysis, elevated liver enzymes, and low platelets count); (5) American Society of Anesthesiologists classification III or higher; or (6) required or asked for general anesthesia.

### Randomization and masking

A biostatistician who was independent of data management and statistical analyses generated random numbers in a 1:1 ratio with a block size of 4 using the SAS 9.2 software (SAS Institute, Cary, NC, USA). The results of randomization were sealed in sequentially numbered opaque envelopes and stored at the site of investigation until shortly before anesthesia.

On the day of cesarean delivery, a designated anesthesia nurse who was otherwise not involved in the trial opened the envelopes according to recruitment sequence, prepared the study drugs (either 0.5 mg/kg ketamine diluted in 100 ml normal saline or 100 ml normal saline) according to the randomization results, and gave the numbered study drugs to the attending anesthesiologists. The allocation codes were then closed in the envelops again until the end of the trial. Therefore, all study participants, attending anesthesiologists, other health-care team members, and outcome assessors were blinded to the study group assignment.

In case of an emergency (such as an unexpected, rapid change in the patient's clinical status), the attending anesthesiologists could request unmasking the treatment allocation, and adjust or interrupt study drug administration when necessary. These situations were recorded and analyses were performed using the intent-to-treat principal.

### Anesthesia and perioperative care

Routine intraoperative monitoring included electrocardiogram, non-invasive blood pressure, and pulse oxygen saturation. Neuraxial anesthesia was performed after establishing intravenous lines with lactated Ringer's solution. The usual practice was combined spinal-epidural anesthesia, which was performed at L2–L3 or L3–L4 interspace. The spinal block was achieved with 10–15 mg of 0.5% ropivacaine; the target block level was T6–T8. An epidural catheter was inserted through the epidural needle for anesthesia maintenance and postoperative analgesia. For patients with indwelling epidural catheter (for labor analgesia), epidural anesthesia was performed with 2% lidocaine and/or 1% ropivacaine at L2–3 interspace. The operating table was tilted 15° to the left when patients were turned to supine position, and vital signs were closely monitored. Vasopressors including ephedrine and phenylephrine were administered to maintain blood pressure; opioids including fentanyl and sufentanil were administered for supplemental analgesia.

Low-segment cesarean delivery was conducted per routine. After clamping the umbilical cord, the study drugs (0.5 mg/kg ketamine for the ketamine group and normal saline for the placebo group) were infused intravenously at a rate of 150 ml/h over a 40-minute period. Study drug infusion continued in the post-anesthesia care unit if not finished during surgery. Patients were monitored for at least 60 min from the end of study drug infusion in the post-anesthesia care unit before being transferred to the postpartum ward. Midazolam could be administered for ketamine-related side effects when considered necessary.

In the general ward, electrocardiogram, non-invasive blood pressure, and pulse oxygen saturation were monitored during the first 6 h after delivery. Postoperative epidural analgesia was provided with a mixture of 0.1% ropivacaine plus 3.33 mg/ml tramadol at a rate of 5 ml/h for 24 h. Mothers and babies were encouraged to stay in the same room and begin breastfeeding early.

### Data collection and outcome assessment

The day before surgery, parturients who were scheduled for elective cesarean delivery were screened with the EPDS after obtaining consents. This is a 10-item questionnaire used to screen perinatal depression; the score ranges from 0 to 30 with higher score indicating more severe depressive symptoms (19). The Chinese version Edinburgh Postnatal Depression Scale has been validated and a cut-off score of 9/10 is recommended for screening depression (20, 21).

For enrolled participants, a standard questionnaire was used to collect baseline data, including demographic variables, previous medical history, history of the present pregnancy, and data of spouses. Level of anxiety was assessed with the Zung Self-Rating Anxiety Scale (score ranges from 20 to 80, with higher score indicating more severe anxiety) (22); level of social support was assessed with the Social Support Rating Scale (score ranges from 11 to 62, with higher score indicating better social support) (23); marital satisfaction was assessed with the ENRICH Marital Satisfaction Scale (score ranges from 10 to 50, with higher score indicating better marital satisfaction) (24). Chinese versions of these scales have been validated (25–27).

Intraoperative data were recorded. Maternal data included the durations of anesthesia and operation, type of anesthesia, fluid infusion, estimated blood loss, and blood transfusion. Neonatal data included sex, birth weight, Apgar Scores at 1 and 5 min after delivery, and requirement of neonatal ward admission. During the period of study drug infusion, non-invasive blood pressure, heart rate, and pulse oxygen saturation were collected every 5 min. At the end of study drug infusion, the level of agitation/sedation was assessed with the Richmond Agitation Sedation Scale, with scores ranging from −5 (unarousable) to 4 (combative) where 0 indicates alert and calm (28).

Our primary endpoint was the depression score at 48 h postpartum, which was assessed with the EPDS during a face-to-face interview by a trained investigator. We chose this primary endpoint because the antidepressant effect of ketamine peaks at 24 h and lasts for about 1 to 2 weeks (10, 11). The secondary outcomes included time to first breast feeding, proportion with breastfeeding within 24 h, duration of neonatal sleep within 24 h, length of hospital stay after childbirth, the score and prevalence of depression at 42 days, and the proportions of maternal and neonatal hospital revisit within 42 days. Depression at 42 days was assessed with the EPDS *via* a telephone interview; a score of ≥10 was defined as having depressive symptoms. Among other outcomes, pain intensity at rest was assessed at 4 h, 8 h, 24 h, and 42 days postpartum with the numeric rating scale (an 11-point scale where 0 indicates no pain and 10 the worst pain). Persistent pain was defined as a numeric rating scale of pain ≥1 that persisted until 42 days. The mode of feeding at 42 days was also recorded.

Adverse events were monitored from the initiation of study drug infusion until the first day postpartum. Potential adverse events included cardiovascular effects such as hypotension (systolic blood pressure <90 mmHg or a decrease of >30% from baseline), hypertension (systolic blood pressure >160 mmHg or an increase of >30% from baseline), bradycardia (heart rate <60 beat per minute or a decrease of >20% from baseline), and tachycardia (heart rate >100 bpm or an increase of >20% from baseline), as well as desaturation (pulse oxygen saturation <90% or an absolute decrease of >5% from baseline) and sedation (a Richmond Agitation Sedation Scale ≤−2). Psychiatric side effects were evaluated and included hallucinations, vivid dreams, dizziness, diplopia, somnolence, hypertonia, and nightmare (29). We also recorded the occurrence of intraoperative and postoperative nausea or vomiting.

### Statistical analysis

#### Sample size estimation

As a pilot trial, we planned to enroll 32 patients in each group. The feasibility objectives of this pilot trial included the following: (1) to refine and test the study protocol for a large multicenter trial; (2) to assess the acceptability of the study protocol to our patients and anesthesiologists; (3) to assess the rate of postpartum depressive symptoms, in order to estimate the sample size and duration of a definitive trial; and (4) to assess the rate of adverse events using the protocol. Feasibility outcomes were recruitment rate, protocol compliance, prevalence of postpartum depressive symptoms at 42 days, and incidence of adverse events.

#### Data analysis

Outcome analyses were primarily performed in the intent-to-treat population; that is, all patients were analyzed in the groups to which they were assigned, excluding those who withdraw consents. For the primary outcome, we also performed analyses in the per-protocol population.

The balance of baseline variables between the two groups were assessed with the absolute standardized differences, calculated as the absolute difference in means, mean ranks, or proportions divided by the pooled standard deviation. Baseline variables with an absolute standardized difference of $\geq 1.96\times \sqrt{\left(n1+n2)/(n1\times n2\right)}$ were considered imbalanced between the two groups and adjusted for in all analyses if considered necessary (30).

As the primary endpoint, depression score at 48 h was compared with Mann-Whitney U test. Median difference and 95% CI were calculated with Hodges-Lehmann estimator. To adjust for the imbalanced baseline variables, we performed generalized linear regression analysis by including age, family income, prepartum hemoglobin, baseline depression score, and baseline Social Support Rating Scale in the model.

For secondary and other outcomes, continuous variables with normal distribution were compared with independent samples t test. Continuous variables with non-normal distribution and discrete variables were compared with Mann-Whitney U test; median differences (and 95% CIs) were calculated with Hodges-Lehmann estimators. Categorical variables were analyzed with chi-square test, continuity-corrected chi-square tests, or Fisher's exact test. Relative risks (and 95% CIs) were calculated. Time-to-event data were analyzed with the Kaplan-Meier survival analysis and log-rank test. Hazard ratios (and 95% CIs) were calculated using the univariable Cox proportional hazard model.

To adjust for the confounding effects of imbalanced baseline variables on depression score at 42 days and presence of depressive symptoms at 42 days, we also performed generalized linear regression and logistic regression analyses, respectively, by including the same factors as above.

For all hypotheses, a two-tailed *P* value of <0.05 was considered statistically significant. Missing data were not replaced. The SPSS 25.0 software (IBM SPSS, Chicago, IL) was used for the statistical analysis.

## Results

### Patient population

**Figure 1:**
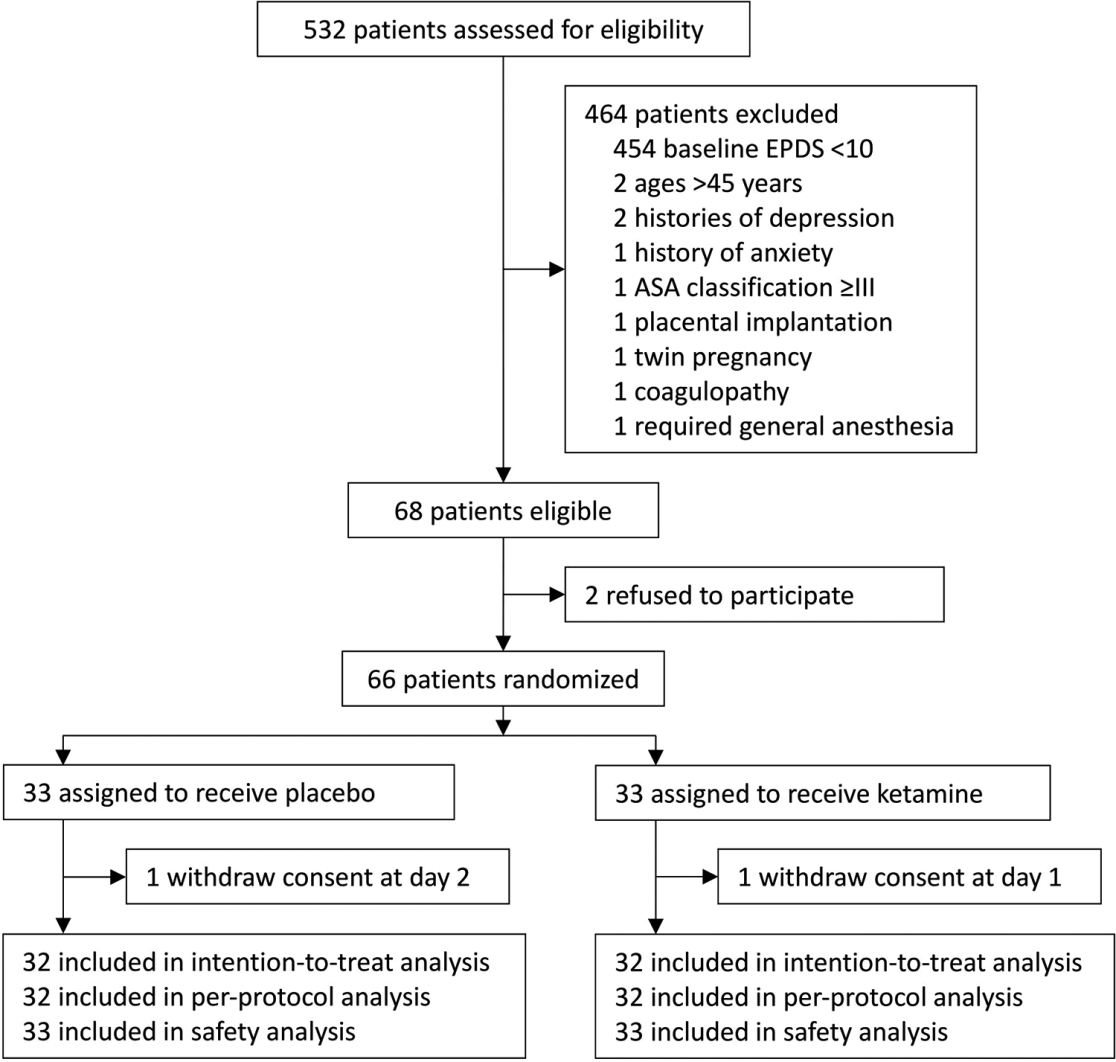
Flowchart of the trial. EPDS, Edinburgh Postnatal Depression Scale; ASA, American Society of Anesthesiologists.

From November 23, 2017 to May 14, 2018, 532 women were assessed for eligibility. Among them, 68 were eligible; 66 were recruited and randomized to receive either ketamine (*n* = 33) or placebo (*n* = 33). During the study period, two parturients withdrew consents during postpartum follow-up (one in each group); there were no protocol violations. As a result, 64 parturients were included in the final intention-to-treat and per-protocol analyses; all 66 parturients were included in the safety analysis (Figure 1). The last patient follow-up was performed on June 25, 2018.

Baseline data were generally balanced between the two groups except that family income was lower and prepartum hemoglobin level was higher in the ketamine group than in the placebo group (Table 1). The sedation score at the end of study drug infusion was lower in women given ketamine, but the difference was not clinically important. Other intraoperative data including those of mothers and neonates were comparable between the two groups (Table 2).

**Table 1:** Baseline data.

Variables	Ketamine (*n* = 32)	Placebo (*n* = 32)	ASD
**Maternal data**			
Age (year)	33 ± 4	35 ± 5	0.453
Body mass index before childbirth (kg/m^2^)	27.4 ± 4.1	27.5 ± 3.0	0.016
Han nationality	28 (87.5%)	30 (93.8%)	0.186
Religious belief^a^	3 (9.4%)	4 (12.5%)	0.106
Education >12 years	25 (78.1%)	27 (84.4%)	0.149
With stable occupation	26 (81.3%)	25 (78.1%)	0.079
Family income (Chinese Yuan/month)			**0** **.** **814**
<10,000	7 (21.9%)	8 (25.0%)	
10,001–20,000	19 (59.4%)	8 (25.0%)	
>20,000	6 (18.8%)	16 (50.0%)	
Covered by social health insurance	28 (87.5%)	25 (78.1%)	0.279
Stressful life events in 2 years^b^	3 (9.4%)	3 (9.4%)	0.000
Pregestational condition			
Medical comorbidity^c^	2 (6.3%)	5 (15.6%)	0.381
Gynecological diseases^d^	9 (28.1%)	6 (18.8%)	0.205
Dysmenorrhea	17 (53.1%)	13 (40.6%)	0.247
Premenstrual syndrome^e^	5 (15.6%)	4 (12.9%)	0.085
History of surgery	15 (46.9%)	19 (59.4%)	0.247
History of adverse pregnancy^f^	18 (56.3%)	17 (53.1%)	0.062
During pregnancy			
Planned pregnancy	21 (65.6%)	18 (56.3%)	0.194
Underwent routine antenatal care	32 (100.0%)	31 (96.9%)	0.044
Attend childbirth classes	26 (81.3%)	27 (84.4%)	0.112
Obstetric diseases^g^	14 (43.8%)	16 (50.0%)	0.124
Low-back pain affecting daily life^h^	12 (37.5%)	14 (43.8%)	0.127
Smoking or alcohol drinking	0 (0.0%)	1 (3.1%)	0.253
Duration of gestation (week)	38.7 (37.9, 39.4)	39.0 (37.9, 39.7)	0.468
Prepartum hemoglobin (g/dl)	12.5 ± 1.2	11.9 ± 1.0	**0** **.** **543**
Depression score (point)^i^	12 (10, 13)	11 (10, 12)	0.186
ENRICH Marital Satisfaction Scale (point)^j^	44 (32, 49)	41 (35, 47)	0.084
Zung Self-Rating Anxiety Scale (point)^k^	39 (35,42)	36 (32, 42)	0.320
Social Support Rating Scale (point)^l^	39 (35, 45)	41 (37, 46)	0.352
**Paternal data**			
Han nationality	30 (93.8%)	31 (96.9%)	0.127
Smoking or alcohol drinking	16 (50.0%)	16 (50.0%)	0.000
Education >12 years	28 (87.5%)	28 (87.5%)	0.000
With stable occupation	32 (100.0%)	30 (93.8%)	0.364

Data are mean ± SD, *n* (%) or median (interquartile range). ASDs in bold indicate >0.490 and were considered imbalanced between the two groups.

ASD, absolute standardized difference.

^a^ Includes Buddhism, Islam, and Christianity.

^b^ Include bereavement, accidental injury, divorce, and/or unemployment.

^c^ Includes asthma, arrhythmia, latent glomerulonephritis, abnormal liver function, and positive hepatitis B surface antigen.

^d^ Include hysteromyoma, ovarian cysts, dysfunctional uterine bleeding, polycystic ovary syndrome, and pelvic inflammatory disease.

^e^ Defined as a consistent pattern of emotional and physical symptoms occurring only during the luteal phase of the menstrual cycle that are of enough severity to interfere with some aspects of life. Diagnosis was made by gynecologists.

^f^ Includes miscarriages, induced abortion, and midtrimester induction of labor due to fetal anomalies.

^g^ Include impaired glucose tolerance/gestational diabetes mellitus, pregnancy-induced hypertension syndrome/preeclampsia, and low free triiodothyronine and/or free thyroxin.

^h^ One of the following activities were affected, including walking, mood, sleep, or concentration, as judged by parturients themselves.

^i^ Assessed with the Edinburgh Postnatal Depression Scale; score ranges from 0 to 30, with higher score indicating more severe depression.

^j^ Score ranges from 10 to 50, with higher score indicating better marital satisfaction.

^k^ Score ranges from 20 to 80, with higher score indicating more severe anxiety.

^l^ Score ranges from 11 to 62, with higher score indicating better social support.

**Table 2:** Intraoperative data.

Variables	Ketamine (*n* = 32)	Placebo (*n* = 32)	*P* value
**Maternal data**			
Duration of anesthesia (min)	90 (75, 112)	89 (73, 106)	0.872
Type of anesthesia			0.196
Epidural	1 (3.1%)	5 (15.6%)	
Combined spinal-epidural	31 (96.9%)	27 (84.4%)	
Fluid infusion (ml)	1,100 (700, 1200)	1,000 (600, 1100)	0.068
Estimated blood loss (ml)	300 (300, 500)	300 (300, 475)	0.632
Blood transfusion^a^	0 (0.0%)	2 (6.3%)	0.492
Duration of operation (min)	53 (41, 68)	52 (39, 66)	0.877
Vasopressors^b^	4 (12.5%)	3 (9.4%)	>0.999
Supplemental opioids	0 (0.0%)	0 (0.0%)	—
Supplemental midazolam	0 (0.0%)	0 (0.0%)	—
Sedation/agitation score at the end of study drug infusion (point)^c^	0 (−1, 0)	0 (0, 0)	0.001
**Neonatal data**			
Male sex	17 (53.1%)	16 (50.0%)	0.802
Consistent with father's preference	27 (84.4%)	28 (87.5%)	>0.999
Consistent with mother's preference	24 (75.0%)	27 (84.4%)	0.351
Birth weight (g)	3344 ± 443	3187 ± 515	0.498
Apgar score			
1 min	10 (10, 10)	10 (10, 10)	0.582
5 min	10 (10, 10)	10 (10, 10)	0.317
Admission to neonatal ward^d^	4 (12.5%)	6 (18.8%)	0.491

Data are mean ± SD, *n* (%) or median (interquartile range).

^a^ Included allogenic blood transfusion and autologous blood salvage and transfusion.

^b^ Included ephedrine and phenylephrine.

^c^ Assessed with the Richmond Agitation Sedation Scale; score ranges from −5 (unarousable) to +4 (combative) and 0 indicates alert and calm (28).

^d^ For the purpose of further monitoring and/or treatment which were considered necessary by the pediatricians. Indications included hypoglycemia, neonatal malformation, neonatal vomiting, and premature delivery.

### Efficacy outcomes

The depression score at 48 h postpartum was median 9 (interquartile range 6 to 13) in the ketamine group and 8 (6 to 10) in the placebo group. There was no significant difference between the two groups (median difference 1, 95% CI −1 to 3, *P* = 0.608; Table 3). After adjustment with predefined factors in a generalized linear regression model, the association between ketamine use and depression score at 48 h was not statistically significant (regression coefficient 0.08, 95% CI −0.11 to 0.27, *P* = 0.422; Table 4). Per-protocol analysis gave the same results.

**Table 3:** Efficacy outcomes.

	Ketamine (*n* = 32)	Placebo (*n* = 32)	Estimated effects (95% CI)^a^	*P* value
**Primary outcome**				
Depression score at 48 h (point)^b^	9 (6, 13)	8 (6, 10)	Median difference = 1 (−1, 3)	0.608
**Secondary outcomes**				
Time to first breastfeeding (h)	24 (1, 48)	7 (1, 48)	Hazard ratio = 0.94 (0.51, 1.74)	0.831
Breastfeeding within 24 h	17 (53.1%)	21 (65.6%)	Relative risk = 0.81 (0.54, 1.22)	0.309
Duration of neonatal sleep within 24 h (h)	20 (18, 22)	20 (18, 22)	Median difference = 0 (−1, 1)	0.885
Length of hospital stay after surgery (day)	4 (3, 4)	3 (3, 5)	Hazard ratio = 0.93 (0.55, 1.56)	0.718
Depression score at 42 days (point)^b^	10 (7, 12)	9 (7, 11)	Median difference = 1 (−1, 2)	0.553
Depression score ≥10 at 42 days	18 (56.3%)	14 (43.8%)	Relative risk = 1.29 (0.78, 2.12)	0.317
Maternal hospital revisit within 42 days^c^	2 (6.3%)	2 (6.3%)	Relative risk = 1.00 (0.15, 6.67)	>0.999
Neonatal hospital revisit within 42 days^d^	2 (6.3%)	5 (15.6%)	Relative risk = 0.40 (0.08, 1.91)	0.426
**Other outcomes**				
Numeric rating scale of pain at rest, point^e^				
At 4 h	3 (2, 3)	4 (3, 4)	Median difference = −1 (−1, 0)	**0** **.** **002**
At 8 h	3 (2, 4)	3 (2, 4)	Median difference = 0 (0, 1)	0.681
At 24 h	4 (3, 5)	4 (3, 4)	Median difference = 0 (0, 1)	0.573
At 42 days	0 (0, 3)	0 (0, 1)	Median difference = 0 (0, 0)	0.133
Persistent pain at 42 days^f^	11 (34.4%)	11 (34.4%)	Relative risk = 1.00 (0.51, 1.97)	>0.999
Exclusive breastfeeding at 42 days	17 (53.1%)	14 (43.8%)	Relative risk = 1.21 (0.73, 2.02)	0.453
**Exploratory analysis**				
Change from baseline depression score (point)				
At 48 h	−4 (−5, 0)	−2 (−4, −1)	Median difference = 0 (−2, 2)	0.908
At 42 days	−2 (−4, 0)	−2 (−5, −1)	Median difference = 0 (−1, 2)	0.705

Data are mean ± SD, *n* (%) or median (interquartile range). *P* values in bold indicate <0.05.

EPDS, Edinburgh Postnatal Depression Scale.

^a^ Calculated as the Ketamine group minus or vs. the Control group.

^b^ Score ranges from 0 to 30, with higher score indicating more severe depression.

^c^ Due to abnormal uterine bleeding, subarachnoid hemorrhage, and cesarean section wound infection.

^d^ Due to jaundice, eczema, and anemia.

^e^ Score ranges from 0 to 10, where 0 = no pain and 10 = the worst pain.

^f^ Defined as the numeric rating scale of pain ≥1 that persisted since childbirth.

**Table 4:** Association between ketamine use and depression scores or depressive score ≥10 after caesarean delivery^a^.

Variables	Unadjusted		Adjusted^b^	
	Regression coefficient or odds ratio (95% CI)	*P* value	Regression coefficient or odds ratio (95% CI)	*P* value
Depression score at 48 h^c^				
Placebo	Ref.		Ref.	
Ketamine	0.07 (−0.10, 0.23)	0.427	0.10 (−0.15, 0.23)	0.675
Depression score at 42 days^c^				
Placebo	Ref.		Ref.	
Ketamine	0.06 (−0.10, 0.22)	0.441	−0.01 (−0.19, 0.18)	0.957
Depression score ≥10 at 42 days^d^				
Placebo	Ref.		Ref.	
Ketamine	1.65 (0.62, 4.44)	0.319	1.02 (0.27, 3.84)	0.974

^a^ Depression was assessed with the Edinburgh Postnatal Depression Scale; score ranges from 0 to 30, with higher score indicating more severe depression.

^b^ Adjusted for age, family income, prepartum hemoglobin, baseline depression score, and baseline Social Support Rating Scale.

^c^ Generalized linear regression analyses.

^d^ Logistic regression analysis.

**Figure 2:**
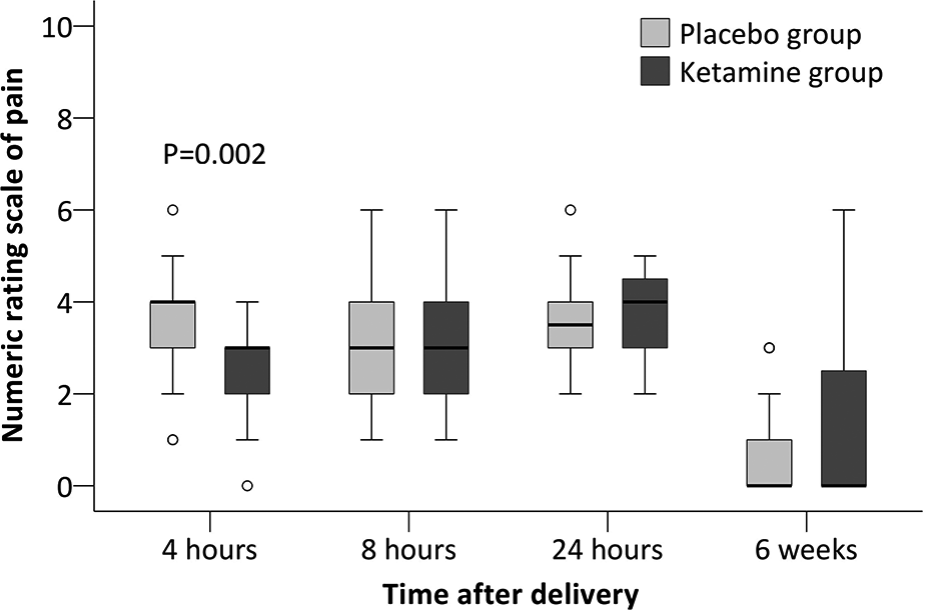
Numeric rating scale of pain at rest after cesarean delivery. The box and whiskers plots show medians, interquartile ranges, and outer ranges; individual circles indicate mild outliers (outside 1.5 times of interquartile range).

Secondary outcomes including time to first breastfeeding, breastfeeding within 24 h, duration of neonatal sleep within 24 h, depression score at 42 days, and presence of depressive symptoms at 42 days did not differ between groups. Among other outcomes, the numeric rating scale of pain was significantly lower in the ketamine group (median difference −1, 95% CI −1 to 0, *P* = 0.002) at 4 h postpartum but not later. The association between ketamine use and depression score at 42 days and presence of depressive symptom at 42 days were not significant after adjustment with factors as above (Tables 3, 4, Figure 2). No women received antidepressant medications within 42 days postpartum.

### Safety outcomes

Intraoperative nausea or vomiting was less common in patients given ketamine (0.0% [0/33] with ketamine vs. 21.2% [7/33] with placebo, *P* = 0.011). Dissociative symptoms, which included hallucination, dizziness, diplopia, and hypertonia, occurred more frequently in the ketamine group (30.3% [10/33] with ketamine vs. 9.1% [3/33] with placebo, *P* = 0.030) but resolved within 60 min after the end of infusion; no treatment was required. Patients given ketamine suffered more sedation at the end of study drug infusion (15.2% [5/33] with ketamine vs. 0.0% [0/33] with placebo, *P* = 0.053), but not statistically significant. Other adverse events including cardiovascular side effects did not differ between groups (Table 5).

**Table 5:** Adverse events.

	Ketamine (*n* = 33)	Placebo (*n* = 33)	*P* value
Intraoperative period			
Hypotension^a^	4 (12.1%)	3 (9.1%)	>0.999
Hypertension^b^	1 (3.0%)	0 (0.0%)	>0.999
Bradycardia^c^	3 (9.1%)	4 (12.1%)	>0.999
Tachycardia^d^	18 (54.5%)	14 (42.4%)	0.325
Desaturation^e^	0 (0.0%)	0 (0.0%)	—
Nausea or vomiting	0 (0.0%)	7 (21.2%)	**0** **.** **011**
Dissociative symptoms	10 (30.3%)	3 (9.1%)	**0** **.** **030**
Hallucination	1 (3.0%)	0 (0.0%)	>0.999
Dizziness	5 (15.2%)	3 (9.1%)	0.708
Diplopia	3 (9.1%)	0 (0.0%)	0.238
Hypertonia	1 (3.0%)	0 (0.0%)	>0.999
Somnolence	4 (12.1%)	0 (0.0%)	0.114
Sedation^f^	5 (15.2%)	0 (0.0%)	0.053
Postoperative period			
Desaturation^e^	0 (0.0%)	0 (0.0%)	—
Nausea or vomiting	7 (21.2%)	5 (15.2%)	0.523
Nightmare	0 (0.0%)	0 (0.0%)	—

Data are *n* (%). *P* values in bold indicate <0.05.

^a^ Defined as systolic blood pressure <90 mm Hg or a decrease of >30% from baseline.

^b^ Defined as systolic blood pressure >160 mm Hg or an increase of >30% from baseline.

^c^ Defined as heart rate <60 beats per minute or a decrease of >20% from baseline.

^d^ Defined as heart rate >100 beats per minute or an increase of >20% from baseline.

^e^ Defined as pulse oxygen saturation less than 90% or a decrease of more than 5% from baseline.

^f^ Defined as a Richmond Agitation Sedation Scale score ≤−2 at the end of study drug infusion.

### Feasibility outcomes

During the study period, 97.1% (66/68) of the eligible patients agreed for study participation. No protocol violation occurred during study drug infusion. The most frequently occurred adverse events during ketamine infusion included dizziness, somnolence, and sedation, but no special treatments were required. One patient in each group withdrew consent during postpartum follow-up, but none of them was due to ketamine-related adverse events. Half of patients with prenatal depressive symptoms (32/64) continued to have depressive symptoms at 42 days.

## Discussion

Results of this pilot trial showed that, in women with prenatal depressive symptoms who were scheduled for cesarean delivery, a single low-dose ketamine infusion did not significantly decrease the depression score at 2 days, but it lowered the incidence of intraoperative nausea and vomiting and the intensity of pain at 4 h following surgery. The acceptability of the intervention, the safety of ketamine infusion, and the prevalence of depressive symptom at 42 days postpartum in this patient population provide valuable data in planning a future definitive study.

As the most widely used screening tool for perinatal depression, the cut-off point of EPDS is debatable and ranges from 10 to 13 in the literature (1, 4). In the present study, we adopted a cut-off point of 10 because it is verified in Chinese women and widely used in clinical practice (21, 31). The etiology of perinatal depression is multifactorial. Identified risk factors include history of psychiatric illness, a family history of depression, previous major depression episodes during pregnancy, poor marital relationship, stressful life events, low socio-economic status, negative attitude towards pregnancy, depression and anxiety during present pregnancy, negative birth experience, poor breastfeeding status, infant illness, and others (32–34). Among these, depression during pregnancy is identified as a strong predictor of postpartum depression (32, 35). According to a review of longitudinal studies, an average of 39% women who experienced antenatal depression continued to have depression after childbirth (36). In line with these, our results showed that 50% of the enrolled patients had depressive symptoms at 42 days. Therefore, women with prenatal depression warrant more attention, further demonstrating the rationality of active intervention in this patient population.

Non-pharmacological measures, such as yoga, exercise, music, massage, acupuncture, and bright light therapy, may be effective to improve prenatal depression but high-quality studies are still lacking (37, 38). Traditional antidepressant medications, such as selective serotonin reuptake inhibitors and serotonin norepinephrine reuptake inhibitors, are used in pregnant women. However, use of these medications is associated increased risk of adverse infant outcomes including miscarriage, congenital cardiac malformations, preterm birth, persistent pulmonary hypertension, and transient neonatal symptoms and is therefore limited to patients with severe or recurrent depression (39, 40).

As a traditional anesthetic/analgesic drug and a newly approved antidepressant (10, 11), low-dose ketamine has been investigated in healthy women undergoing cesarean delivery. However, regarding its effect in women with prenatal depression, conclusion cannot be reached according to available evidences due to low-risk patients (only a small proportion had prenatal depression) and insufficient sample size (16–18). In the present study, we did not find that low-dose ketamine could decrease the depression score at 2 days postpartum. This is understandable considering the small sample size in our pilot trial and the multi-etiology of perinatal depression (4, 33, 34). As suggested by Devereaux et al. (41), it is only realistic to expect a moderate treatment effect when an intervention is targeting a disease with multifactorial etiology. Therefore, the therapeutic effect of low-dose ketamine in women with prenatal depression needs to be clarified in a large sample size trial.

Our results showed that intraoperative nausea or vomiting occurred less frequently in the ketamine group patients. In a previous trial comparing propofol-ketamine combination vs. propofol alone for colonoscopy, nausea/vomiting was similarly less common with ketamine (42). In other studies, ketamine supplemented analgesia reduced postoperative nausea and vomiting by reducing opioid consumption (43). We also found that intraoperative low-dose ketamine significantly reduced pain severity at 4 h after surgery but not thereafter. Similar result was reported by others. In a meta-analysis of randomized trials, intraoperative ketamine significantly lowered pain score at 2 h but not at 24 h following cesarean delivery under spinal anesthesia (43).

We note that the proportions of both breastfeeding within 24 h and exclusive breastfeeding at 42 days were lower in the present trial than in our previous study (59.4% vs. 80.6% within 24 h and 48.4% vs. 68.8% at 42 days, respectively) (31). This can be explained by the fact that both the prenatal depression score (median 11 [interquartile range 10 to 13] vs. 6 [5 to 8]) and the prevalence of depressive symptom at 42 days (50.0% vs. 17.3%) were higher in our patients than in those of the previous study (31). Available evidence suggests that perinatal depressive symptoms are negatively associated with breastfeeding exclusivity and duration (44). Less breastfeeding may produce negative effects on children's development and even mortality (45, 46).

This pilot trial had a high recruitment rate [97.0% (66/68)], indicating a good acceptance by the potential participants. The study protocol was easy to implement and well accepted by attending anesthesiologists, nurses, and investigators. The most frequent ketamine-related symptoms included dizziness, somnolence, and sedation. Dissociative symptoms occurred in a third of our patients given ketamine but were well tolerated; no special treatment was required. Other trials also reported high prevalent but tolerable ketamine-related side effects (16, 47). Finally, 50% of our patients with prenatal depressive symptoms continued to have depression at 42 days; this provides an important clue for sample size estimation in our future trial. However, none of them required antidepressant medications within 42 days.

There are some limitations. First, as a pilot trail, the sample size was not sufficient to detect difference in depression score at 2 days postpartum. Second, depressive symptoms were not evaluated by psychiatrists. However, the EPDS is a widely used screening tool for both prenatal and postpartum depression by trained raters, and have been validated in clinical studies (21). Third, some potential risk factors of postpartum depression were not collected, such as the oxytocin exposure during labor and the sleep quality after childbirth (33, 48). Future studies should consider these methodological issues.

## Conclusion

Our pilot trial showed that, in women with prenatal depressive symptoms, a single low-dose ketamine infusion did not decrease depression score at 2 days; however, it reduced intraoperative nausea or vomiting and relieved pain intensity early after cesarean delivery. The trial protocol is feasible for a large randomized trial investigating the effect of low-dose ketamine in women with pregnancy depression.

## Data Availability

The raw data supporting the conclusions of this article will be made available by the authors, without undue reservation.
